# Subdividing Y-chromosome haplogroup R1a1 reveals Norse Viking dispersal lineages in Britain

**DOI:** 10.1038/s41431-020-00747-z

**Published:** 2020-11-02

**Authors:** Gurdeep Matharu Lall, Maarten H. D. Larmuseau, Jon H. Wetton, Chiara Batini, Pille Hallast, Tunde I. Huszar, Daniel Zadik, Sigurd Aase, Tina Baker, Patricia Balaresque, Walter Bodmer, Anders D. Børglum, Peter de Knijff, Hayley Dunn, Stephen E. Harding, Harald Løvvik, Berit Myhre Dupuy, Horolma Pamjav, Andreas O. Tillmar, Maciej Tomaszewski, Chris Tyler-Smith, Marta Pereira Verdugo, Bruce Winney, Pragya Vohra, Joanna Story, Turi E. King, Mark A. Jobling

**Affiliations:** 1 Department of Genetics & Genome Biology, University of Leicester, Leicester, UK; 2 Department of Human Genetics, KU Leuven—University of Leuven, Leuven, Belgium; 3 Laboratory of Socioecology and Social Evolution, KU Leuven—University of Leuven, Leuven, Belgium; 4 Histories vzw, Zoutwerf 5, 2800 Mechelen, Belgium; 5 School of History, Politics and International Relations, University of Leicester, Leicester, UK; 6 Postboks 420, 5501 Haugesund, Norway; 7 UMR5288, Laboratoire d’Anthropologie Moléculaire et Imagerie de Synthèse, Université Paul Sabatier, Toulouse, France; 8 Department of Oncology, University of Oxford, Oxford, UK; 9 Department of Biomedicine & Centre for Integrative Sequencing, Aarhus University, Aarhus, Denmark; 10 Department of Human Genetics, Leiden University Medical Centre, Leiden, The Netherlands; 11 School of Archaeology and Ancient History, University of Leicester, Leicester, UK; 12 National Centre for Macromolecular Hydrodynamics, University of Nottingham, Sutton Bonington Campus, Loughborough, UK; 13 Museum of Cultural History, University of Oslo, Oslo, Norway; 14 Lille Borgenveien 2B, 0370 Oslo, Norway; 15 Division of Forensic Sciences, Norwegian Institute of Public Health, Oslo, Norway; 16 Hungarian Institute for Forensic Sciences, Institute of Forensic Genetics, Budapest, Hungary; 17 Department of Forensic Genetics and Forensic Toxicology, National Board of Forensic Medicine, Linköping, Sweden; 18 Division of Cardiovascular Sciences, Faculty of Biology, Medicine and Health, University of Manchester, Manchester, UK; 19 Division of Medicine and Manchester Academic Health Science Centre, Manchester University NHS Foundation Trust Manchester, Manchester, UK; 20 Department of History, University of York, Heslington, York, UK; 21 Present Address: Department of Health Sciences, University of Leicester, University Road, Leicester, LE1 7RH UK; 22 Present Address: Wellcome Sanger Institute, Hinxton, Cambridge, UK; 23 Present Address: Institute of Biomedicine and Translational Medicine, University of Tartu, Tartu, 50411 Estonia; 24 Present Address: Centre for Genetics and Genomics, University of Nottingham, Queen’s Medical Centre, Nottingham, UK; 25 Present Address: MRC Human Genetics Unit, MRC IGMM, University of Edinburgh, Western General Hospital, Edinburgh, UK; 26 Present Address: Smurfit Institute of Genetics, Trinity College, Dublin 2, Ireland

**Keywords:** Genetic variation, Haplotypes, Genetic markers

## Abstract

The influence of Viking-Age migrants to the British Isles is obvious in archaeological and place-names evidence, but their demographic impact has been unclear. Autosomal genetic analyses support Norse Viking contributions to parts of Britain, but show no signal corresponding to the Danelaw, the region under Scandinavian administrative control from the ninth to eleventh centuries. Y-chromosome haplogroup R1a1 has been considered as a possible marker for Viking migrations because of its high frequency in peninsular Scandinavia (Norway and Sweden). Here we select ten Y-SNPs to discriminate informatively among hg R1a1 sub-haplogroups in Europe, analyse these in 619 hg R1a1 Y chromosomes including 163 from the British Isles, and also type 23 short-tandem repeats (Y-STRs) to assess internal diversity. We find three specifically Western-European sub-haplogroups, two of which predominate in Norway and Sweden, and are also found in Britain; star-like features in the STR networks of these lineages indicate histories of expansion. We ask whether geographical distributions of hg R1a1 overall, and of the two sub-lineages in particular, correlate with regions of Scandinavian influence within Britain. Neither shows any frequency difference between regions that have higher (≥10%) or lower autosomal contributions from Norway and Sweden, but both are significantly overrepresented in the region corresponding to the Danelaw. These differences between autosomal and Y-chromosomal histories suggest either male-specific contribution, or the influence of patrilocality. Comparison of modern DNA with recently available ancient DNA data supports the interpretation that two sub-lineages of hg R1a1 spread with the Vikings from peninsular Scandinavia.

## Introduction

**Fig. 1:**
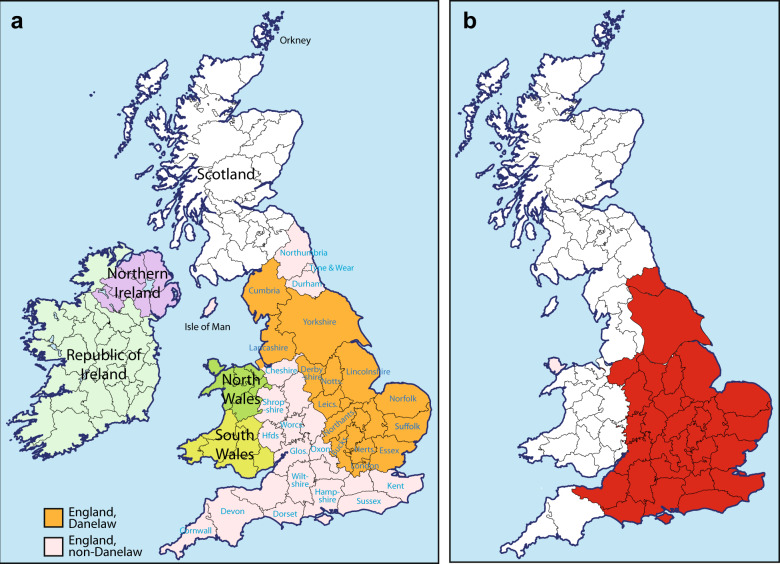
Map of Britain and Ireland showing the Danelaw, the regions sampled, and the predominant PoBI autosomal cluster. **a** English counties (named in blue font) and regions studied here, and the extent of the Danelaw, the region under Scandinavian control from the late ninth to the late eleventh century; **b** Extent (in red) of the predominant cluster revealed by FineSTRUCTURE analysis in the Leslie et al. study [8] of autosomal SNP data in the PoBI samples (referred to there as ‘Central/South England’). Bucks. Buckinghamshire; Glos. Gloucestershire; Herts. Hertfordshire; Hfds. Herefordshire; Leics. Leicestershire; Northants. Northamptonshire; Notts. Nottinghamshire; Oxon Oxfordshire. Map images modified from an original work by Wikishire, CC BY-SA 4.0, https://commons.wikimedia.org/w/index.php?curid=36830415.

The influence of Viking-age Scandinavian migrations on the British Isles is abundantly demonstrated by archaeological finds [1] and linguistic evidence embedded in place names [2]. ‘Danelaw’ is an early eleventh century term for the part of northern and eastern England that came under the control of various Scandinavian rulers following a peace treaty agreed by the West Saxon king Alfred (d. 899 CE), and the Viking leader Guthrum (d. 890 CE) (Fig. 1a). It shows a high density of contemporary Scandinavian metalwork items [3], and high proportions of Scandinavian major and minor place names [2]. This pervasive Scandinavian cultural influence has been argued to indicate the impact of substantial numbers of Viking settlers [4], not just local cultural shift under an incoming elite. Despite its name, the Danelaw presents good evidence of a variety of Scandinavian influences, not restricted to the modern category of Danes. This is reflected in place names (such as Normanton and Normanby) that contain Old West Norse (corresponding to modern Norwegian) forms [5], as well as in finds of artefacts typical of the Irish Sea region, including, for example, stone sculpture in Derbyshire [6].

Genetic studies of modern populations can illuminate the demographic impacts of past migrations [7], and autosomal single-nucleotide polymorphism (SNP) data have provided insights into Scandinavian contributions in both Britain [8] and Ireland [9, 10]. Analysis of data from the ‘People of the British Isles’ (PoBI) cohort [8] defined 17 genetic clusters showing geographically differentiated patterns. Ancestry profiles were generated for these clusters, based on estimated contributions from continental European sources. The dominant cluster in Orkney (part of the Kingdom of Norway for 600 years) showed 25% Norwegian ancestry, taken to reflect past Norse Viking contributions; relatively high Norwegian ancestry was also inferred in western Britain and eastern areas that once comprised the early medieval kingdom of Northumbria. The most prominent cluster, including about half of the sample, covered most of southeast and central England, stretching up the east coast (Fig. 1b); this cluster encompassed the southern limit of the Danelaw, and was therefore taken as evidence for limited Viking input to eastern England. The cluster’s estimated ~35% ancestry from north-west Germany was ascribed to earlier ‘Saxon’ migrations. This interpretation has been challenged [4], partly on the grounds that north-west German contributions may equally reflect Danish Viking influence; in this view, the lack of a ‘Danelaw signal’ could result from free migration within lowland Britain over the last millennium. A similar SNP-based study of Ireland [10] indicated a major influence from northern Europe and Scandinavia, particularly in eastern Ireland, interpreted as a Norse Viking contribution and compatible with attested patterns of settlement, including the Viking foundation of Dublin.

This autosomal picture of a major Viking contribution in Ireland contrasts with previous studies [11] of the male-specific region of the Y chromosome (MSY), which found no evidence of Scandinavian input, even among men carrying Irish surnames derived from Old Norse. This could either suggest improbably female-biased Viking contribution, loss through genetic drift, or some post-Viking-age sociocultural bias against men who had Scandinavian paternal ancestry. By contrast, prior studies of paternal and maternal Scandinavian ancestry in Shetland, Orkney, the Western Isles and Iceland [12] found evidence of paternal Viking contributions, increasing with distance from the Scandinavian homeland.

Within north-western Europe, peninsular Scandinavia (Norway and Sweden) shows the highest frequencies of one MSY lineage, haplogroup (hg) R1a1 (26% in Norway [13], and 12% in Sweden [14]). The finding of the same haplogroup in the North Atlantic islands of Orkney (20% [15]), the Faroes (52% [16]) and Iceland (24% [17]), as well as in Greenland (9% [18]), with their well-evidenced histories of Viking settlement, led to the idea of hg R1a1 as a signature of recent male Scandinavian migration. This gains some support from the finding that the English regions of West Lancashire and the Wirral Peninsula (the north-western part of Cheshire [Fig. 1a]), settled by Vikings in 902 CE, show significantly elevated proportions of hg R1a1 when samples are ascertained using local medieval surnames to minimise the effect of recent immigration [19]. The idea of hg R1a1 as a ‘Viking marker’ has entered the popular imagination in books about genetic history [20, 21], one of which [20] goes so far as to label this haplogroup as ‘the clan of Sigurd’. However, the question of whether the presence of this haplogroup in the British Isles and other parts of north-western Europe signifies Viking migration remains unanswered, and could be addressed by subdivision of R1a1 using a larger number of SNPs.

The first two attempts to sub-divide hg R1a1 [22, 23] focused on the relationships [23] between European and Asian sub-haplogroups. A more recent study resequenced almost 500 hg R1a1 chromosomes, but concentrated on the history of sub-lineages among Ashkenazi Levite Jews [24]. None of these studies has addressed the question of the possible Viking origin of R1a1 sub-haplogroups.

Previously, we generated extensive MSY sequence data in each of 448 human males [25]. These included samples from Norway, Orkney, England and Denmark, giving a total of 27 hg R1a1 Y chromosomes, in which many novel sequence variants were ascertained. Here we exploit this resource to further investigate R1a1 sub-haplogroups in Scandinavia and western Europe. We compare the frequency of R1a1 and its sub-lineages with regions showing lower and higher Scandinavian autosomal contribution estimated in the PoBI cohort [8] and with the Danelaw, and investigate the expansion histories of sub-lineages using multiple short-tandem repeats (STRs).

## Materials and methods

### Samples

By surveying DNA samples from a total of 10,338 males, we identified 1252 carrying hg R1a1 Y chromosomes (Supplementary text; Tables S1 and S2). These belonged to 42 populations (35 European), and include 138 hg R1a1 samples from England (total 2411, including samples from the PoBI cohort [8] and other published studies [26–28], as well as newly sampled individuals) that were divided into sub-regions based on counties; some adjacent counties with small sample sizes were merged (Table S1). All new samples were ascertained based on birthplace of paternal grandfather and recruited with written informed consent. Not all identified hg R1a1 samples were sub-typed; some DNAs were unavailable, and we represented the large Polish, Bhutanese and Nepalese sets by random sub-samples (Table S1). The total number sub-typed was 619.

### SNP typing

**Fig. 2:**
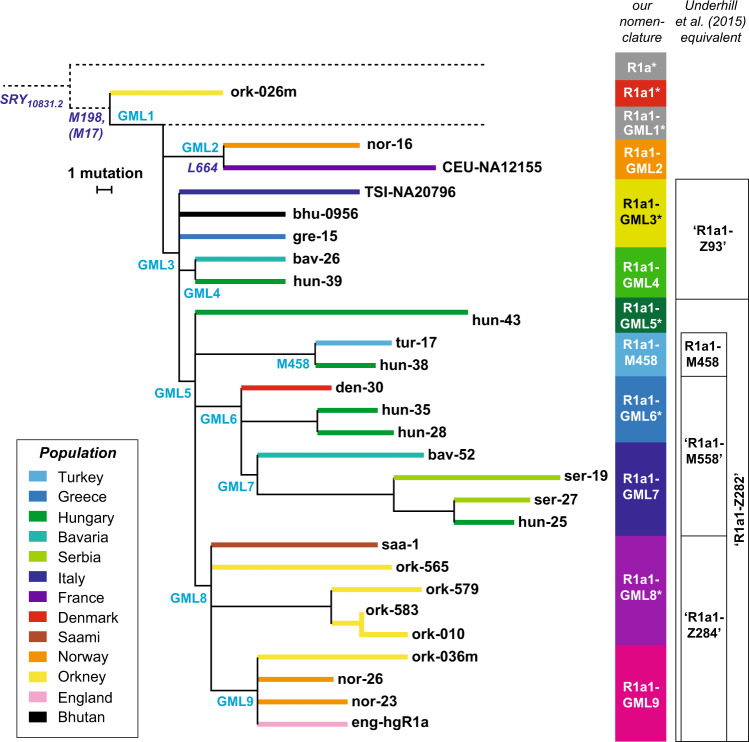
Subdivision of haplogroup R1a1 based on sequencing data. Phylogeny [25] including 27 different MSY sequences, with branch lengths proportional to the number of single-nucleotide mutations. Terminal branches are coloured by population, as shown in the key, and sample names are given at the tips of branches. SNPs chosen for typing in this study are indicated on relevant branches in plain text. Other mutations (e.g., L664) are in italics, and were not typed in all samples. To the right is indicated the approximate equivalence to lineages previously identified [23, 34], and verified in this study. Nomenclature of R1a and R1a1 here is according to ref. [50]. Unobserved lineages, including the deep-rooting paragroup R1a1-GML1*, are indicated by dotted lines.

We chose nine SNPs [25] from the phylogeny (Fig. 2) to analyse in the hg R1a1 sample set (named here GML1: rs541419267; GML2: rs747137438; GML3: rs112157633; GML4: rs778296366; GML5: rs112563127; GML6: rs556726425; GML7: rs770125881; GML8: rs765730048; GML9: rs761494431), adding the SNP M458 identified in an earlier study [29]. A SNaPshot (Applied Biosystems) minisequencing multiplex (Supplementary text; Table S3) was designed to type these ten SNPs and applied to the 619 haplogroup R1a1 samples. Following PCR and single-base primer extension, products were analysed on an ABI3130xL Genetic Analyser (Applied Biosystems). To relate the chosen markers to the phylogeny published previously [23], we assayed six additional SNPs (M558, Z95, Z280, Z284, Z93, M417; Supplementary text) in selected samples.

### Y-STR typing

Twenty-three Y-STRs (DYS19, DYS389I, DYS389II, DYS390, DYS391, DYS392, DYS393, DYS385ab, DYS437, DYS438, DYS439, DYS448, DYS456, DYS458, DYS635, YGATAH4, DYS481, DYS533, DYS549, DYS570, DYS576 and DYS643) were amplified in all 619 samples using the PowerPlex Y23 system (PPY23, Promega Corporation, Madison, WI), following the manufacturer’s instructions, separated on an ABI3130xL and analysed using GeneMapper v 4.0 software (both Applied Biosystems).

### Data analysis

Y-STR haplotype relationships were displayed using median-joining networks [30] based on 21 STRs, using Network 5.0 and Network Publisher (www.fluxus-engineering.com/sharenet.htm). Time to most recent common ancestor (TMRCA) was estimated using average square distance, the mean pedigree mutation rate (3.751 ± 0.694 × 10^−3^/STR/generation; www.yhrd.org), and a generation time of 30 years [31]. We also tested the effect of using subsets of Y-STRs with ‘slow’ and ‘fast’ mutation rates. Further details on network construction and dating are in Supplementary text.

Population differentiation tests and comparisons based on mean per locus diversity from STR data were carried out in Arlequin 3.5 [32].

Comparison of Y-chromosomal haplogroup distributions with regions in Britain showing high levels of Norwegian plus Swedish autosomal contributions [8], was done as described in Supplementary text and Table S4, and tested via a chi square test. Comparisons of Y-chromosomal lineages were also done between ‘Danelaw’ and ‘non-Danelaw’ regions (Table S4).

To compare populations with respect to their R1a1 sub-haplogroup frequencies, principal coordinates analysis was performed using GenAlEx [33].

## Results

Previously [25], we generated 3.7 Mb of MSY sequence data in each of 448 human males. These included 334 randomly sampled individuals from 17 populations from Europe and the Middle East, plus individuals chosen because their Y chromosomes belonged to specific lineages. The resulting phylogeny included 27 MSY sequences within haplogroup R1a1 (Fig. 2).

To place these within the context of previous analyses of R1a1 sub-haplogroups, we compared the phylogeny with published data [23, 34]. We chose six published SNPs not included in our sequenced MSY regions [23] and typed them in the 27 samples sequenced in our study [25]. In addition, our dataset contained the male CEU-NA12155, allowing us to use 1000 Genomes Project data to infer the position in our phylogeny of the variant CTS4385 (ref. [34]). This showed that, as expected, our samples include a limited representation and resolution of Asian R1a1 sub-haplogroups [23], but a good representation of sub-haplogroups within Europe (Fig. 2). We also observe a deep-rooting western-European lineage (R1a1-GML2, probably synonymous with CTS4385) that was previously [23] undefined.

To explore the distribution of the R1a1 sub-haplogroups within larger samples, we selected ten SNPs for analysis in a PCR minisequencing multiplex. To maximise informativeness, each SNP was chosen on the basis that in the 27 sequenced chromosomes [25] its derived state defined a European sub-haplogroup encompassing at least two individuals belonging to different populations.

**Fig. 3:**
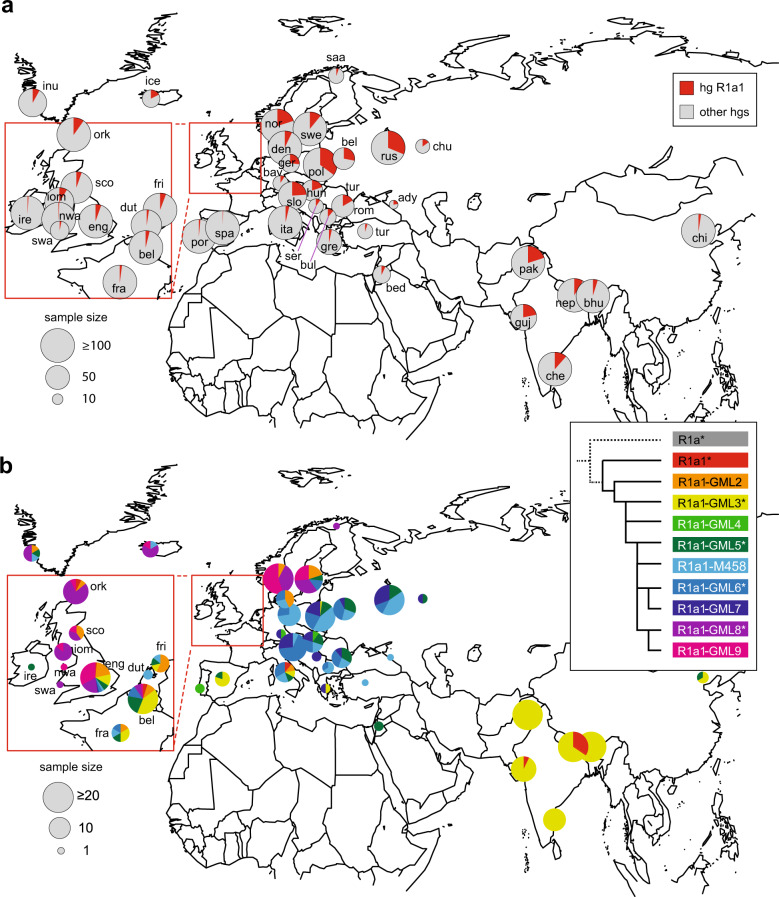
Geographical distributions of haplogroup R1a1 and its sub-haplogroups. **a** Distribution of haplogroup R1a1 in the analysed samples. Pie-charts indicate populations, with area proportional to sample size up to 100, as indicated in the key, and the red sector showing the proportion of hg R1a1. **b** Distribution of sub-haplogroups of R1a1. Populations are represented by pie-charts with area proportional to hg R1a1 sample size up to 20, as indicated in the key, and sectors indicating sub-haplogroup frequencies within R1a1, according to the colour-coded phylogeny top right. Populations in the British Isles and surroundings are labelled as follows: ork Orcadian; sco Scottish mainland; eng English; ire Irish; iom Isle of Man; nwa North Wales; swa South Wales; fre French; bel Belgian; dut Dutch; fra French; fri Frisian; other populations are: ady Adygei; bav Bavarian; bed Bedouin; bel Belarusian; bhu Bhutanese; bul Bulgarian; che Chennai; chi Chinese; chu Chuvash; den Danish; ger German; gre Greek; guj Gujarati; hun Hungarian; ice Icelandic; inu Inuit; nep Nepalese; nor Norwegian; pak Pakistani; pol Polish; por Portuguese; rom Romanian; rus Russian; saa Saami; ser Serbian; slo Slovenian; spa Spanish; swe Swedish; tur Turkish. Additional information about the frequency distributions of sub-haplogroups can be found in Tables S1 and S5.

To provide a larger set of haplogroup R1a1 chromosomes for sub-haplogroup analysis, we then surveyed a total of 10,338 males with existing Y-chromosome data, identifying a total of 1252 males carrying hg R1a1 Y chromosomes. Of these, 1067 (from 36 populations) were European, while 187 (from 7 populations) were Asian. Observed sample frequencies of R1a1 (Table S1; Fig. 3a) are consistent with previous results [29]: high frequencies are seen in central Europe (e.g., >40% in Poland and Russia), India (28% in Gujarat) and Norway (25%). The frequency in our large English sample is 6% (138/2411). Given the low frequencies of hg R1a1 in most western-European populations, many per-population sample sizes are small, limiting the power to test the significance of frequency differences among groups.

### Geographical distribution of hg R1a1 sub-lineages

The SNP multiplex was typed on 619 DNA samples from our hg R1a1 collection (Fig. 3b; Tables S1, S4; Supplementary text). The results can be visualised in interactive form online via the Microreact tool [35] at https://microreact.org/project/P2bq1LnPR. Based on the phylogeny (Fig. 2), the typed SNPs define a maximum of 11 sub-haplogroups; of these, we find 10 (one deep-rooting paragroup, R1a1-GML1*, is not observed). The frequencies of the observed sub-haplogroups vary greatly in different populations (Fig. 3b; Table S5). As expected, given the European bias governing our SNP choices, most of the Asian samples in our dataset belong to the paragroup R1a1-GML3*—equivalent to the clade defined [23] by the SNP Z93 (Fig. 2). We do not address these any further here.

The European samples display a much greater variety of the distinguishable sub-haplogroups, and with strong geographical structuring that is consistent with the distribution suggested by the phylogeny (Fig. 2). Central Europe is dominated by sub-haplogroups R1a1-GML5*, R1a1-GML6*, R1a1-GML7 and R1a1-M458 (blue and green colours in Fig. 3b), but in the British Isles, Iceland, Norway and Sweden the sub-haplogroups R1a1-GML8* and R1a1-GML9 predominate (purple and magenta; 70% of hg R1a1; *n* = 256). These sub-lineages are relatively rare in continental Europe (5% of hg R1a1; *n* = 79) and are absent from our Danish sample. The sub-haplogroup R1a1-GML2 (orange) is found widely, though at low frequency, throughout western Europe; most Danish hg R1a1 chromosomes belong to this sub-haplogroup, and it also comprises 8% of hg R1a1 in Norway, and 20% of hg R1a1 in Sweden. Interestingly, R1a1-GML2 lies phylogenetically basal to the previously defined Asian-European split [23], yet is absent from our Asian samples, and also from central and eastern Europe. European examples of chromosomes belonging to the paragroup R1a1-GML3* are also observed (including in the British Isles), but not in central Europe or in Scandinavia.

The proportions of hg R1a1 chromosomes belonging to R1a1-GML8* and R1a1-GML9 are 21% and 33%, respectively, in Great Britain (the island containing England, Wales and mainland Scotland), and 37% and 55% in Norway. In Orkney and the Isle of Man, by contrast, the paragroup R1a1-GML8* predominates significantly (86% and 79% of hg R1a1 respectively; both with *p* value <0.001 cf Britain; chi square test). This may reflect different sources of migrants compared to the mainland, and/or genetic drift in island populations. To illuminate this, we examined the sub-regional frequencies of these two lineages within Norway, comparing the inland regions (Oppland and Hedmark), with northern coastal (Trondheim, Møre og Romsdal and Nord-Trøndelag), and southern and western coastal regions (Bergen, Stavanger, Sogn og Fjordane, Hordaland and Rogaland). Sub-haplogroup R1a1-GML9 is not significantly different in frequency between these areas. However, R1a1-GML8* is significantly overrepresented in the inland and northern coastal regions compared to the southern/western coastal regions (*p* value = 0.006).

### STR-based analysis of hg R1a1 sub-lineages

To examine diversity within the hg R1a1 sub-haplogroups, we typed 23 Y-STRs in all chromosomes (Table S2). Based on these data alone, there is little population sub-structure or power to predict SNP sub-haplogroups (Fig. S1), as has been suggested before for smaller numbers of Y-STRs [23].

**Fig. 4:**
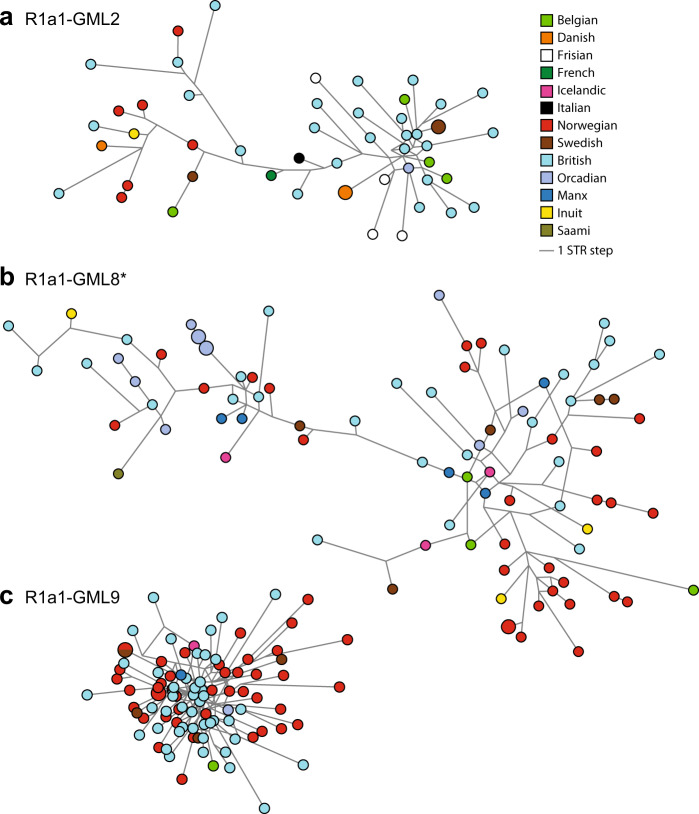
Median-joining networks for three sub-haplogroups of haplogroup R1a1. Networks (all at the same scale) based on 21 Y-STRs for (**a**) R1a1-GML2; (**b**) paragroup R1a1-GML8*; (**c**) R1a1-GML9. Circles represent haplotypes, with areas proportional to frequency, and coloured to represent populations as shown in the key. Lines between haplotypes (links) represent STR mutational steps.

In order to address possible Scandinavian migration to Britain in the early middle ages, we focused on the sub-haplogroups frequent in peninsular Scandinavia, Iceland and the British Isles (R1a1-GML2, R1a1-GML8* and R1a1-GML9). We constructed median-joining networks for all three (Fig. 4) to ask if the relationships among STR haplotypes supported the idea of a Viking-Age spread. We estimated TMRCA for R1a1-GML2 and R1a1-GML9, and also for R1a1-GML8 (encompassing the sub-lineage R1a1-GML9), though it is important to note that these estimates are expected to pre-date any migration event of interest. While TMRCA estimates presented here for R1a1-GML8 and R1a1-GML9 are robust to the choice of Y-STRs and their different mutation rates (Supplementary text), that for R1a1-GML2 is significantly older when the 11 slow-mutating STRs are used for dating (Fig. S2). Date estimates given below are those based on 21 Y-STRs.

(1) The network for R1a1-GML2 (Fig. 4a) includes a star-like structure (right-hand part) that may indicate expansion, and contains mostly British haplotypes, with some examples from Denmark, Sweden, Friesland and Belgium, but lacks any Norwegian haplotypes; the rest of the network (left-hand part) is more extended and includes just six Norwegian and seven British haplotypes. TMRCA is estimated as 3202 years ago (2589-3815 YA).

(2) The network for the R1a1-GML8* paragroup (Fig. 4b) presents an extended structure suggesting the presence of undefined sub-lineages. At least three different clusters can be identified, each of which contains haplotypes from peninsular Scandinavia as well as the British Isles. This sub-lineage contains the majority of Manx and Orkney haplotypes, most of the latter (8/11) clustering together, suggesting a possible founder effect. The TMRCA of the R1a1-GML8 clade, which includes both the R1a1-GML8* paragroup and the R1a1-GML9 sub-haplogroup, is 3246 YA (2624-3868 YA).

(3) The network for R1a1-GML9 (Fig. 4c) contains mostly Norwegian and British haplotypes and is condensed and star-like, suggesting recent population expansion [36]. For the network as a whole, TMRCA is 2273 YA (1838–2708 YA). In a population differentiation test based on haplotype frequencies the British and Norwegian + Swedish samples are not significantly different, but the average gene diversity over loci is lower in the British than the Scandinavian sample, albeit not significantly so (0.271 ± 0.145 vs 0.318 ± 0.168).

The shapes of these networks and the geographical distributions of haplotypes suggest the R1a1-GML8 clade and its included sub-haplogroup R1a1-GML9 as candidate Viking lineages that spread to the British Isles. It would seem worthwhile to estimate a split time between the British and Scandinavian groups of R1a1-GML8 chromosomes, but in fact these groups are very similar in their range of haplotypes (in AMOVA analysis, 98% of the variance is within-group, and only 2% between), and their modal haplotypes are identical, making a split-time calculation uninformative. What this similarity does suggest, however, is that any contribution of lineages from Scandinavia to Britain must have been sufficiently large to avoid a strong founder effect.

### Geographical differentiation of sub-lineages within Britain

To further address the question of Viking migration, we asked whether the geographical distributions of hg R1a1 overall, and the candidate Viking sub-lineages R1a1-GML8* and R1a1-GML9 in particular, correlated with regions of Scandinavian influence within Great Britain (Table S4). For this we considered two different subdivisions (Table S4): (1) regions showing a signal of higher (≥10%) autosomal contribution from Norway and Sweden in the PoBI study [8], versus regions showing lower (<10%) contribution, and (2) the Danelaw (Fig. 1a) versus the rest of Britain. We see no significant difference between regions with high vs low Norwegian + Swedish autosomal ancestry proportions for either the frequency of hg R1a1 as a whole, or the two candidate Viking sub-lineages (hg R1a1: 5.5% vs 5.3%; *p* = 0.869, and R1a1-GML8* + R1a1-GML9: 3.1% vs 2.5% *p* = 0.319, respectively). However, a significantly higher frequency of hg R1a1 is observed in the Danelaw than in the rest of Britain (6.6% vs 4.2%; *p* value = 0.006), and a marginally significantly higher frequency of R1a1-GML8 (including its sub-lineage R1a1-GML9) is also seen (3.5% vs 2.2%; *p* value = 0.040).

**Fig. 5:**
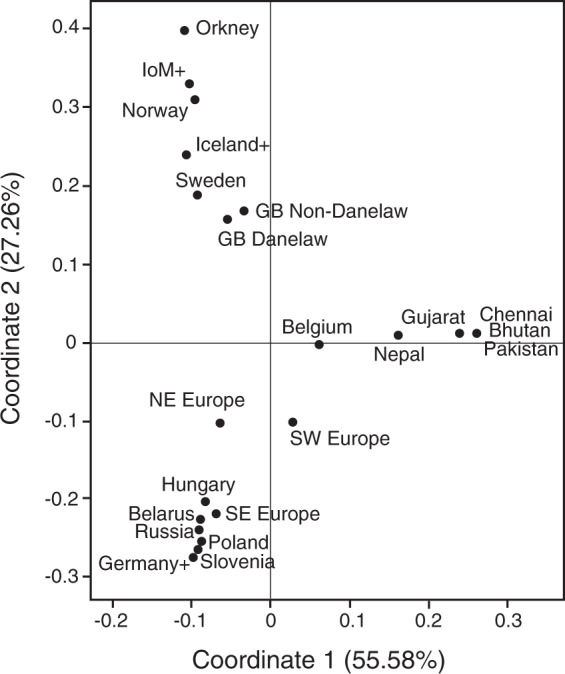
Principal coordinates analysis of populations based on hg R1a1 sub-haplogroup frequencies. The minimum population size is 10, and pooling was necessary to achieve this in some cases, as follows: IoM+ = Isle of Man, Scotland and Ireland; Germany+ = Germany and Bavaria; Iceland+ = Iceland and Inuit; SW Europe = France, Spain, Portugal and Italy; SE Europe = Bulgaria, Greece, Turkey, Romania and Serbia; NW Europe = Frisia, Netherlands and Denmark. The two ‘GB’ populations omit the Scottish samples. For some populations, sample size was too small for inclusion and reasonable pooling was not possible.

Figure 5 shows a principal coordinates analysis plot of populations based on sub-haplogroup frequencies. This confirms the general similarity of populations from Britain and Peninsular Scandinavia, and confirms the closer Scandinavian affiliation of the Danelaw compared to the non-Danelaw subdivision of Britain. Given that Y-STR haplotypes fail to resolve haplogroups (Fig. S1) a similar analysis based on these haplotypes is not expected to give a coherent picture of population relationships, and indeed this is so (Fig. S3).

## Discussion

Haplogroup R1a1 represents one of Eurasia’s major patrilineages [37], and has been considered unusual because of its high frequency both in Asia (the Indian subcontinent in particular [38]) and in central Europe and the Scandinavian peninsula [29]. The emphasis of previous studies of this haplogroup has been on Central and Eastern Europe and Asia [22–24]. However, our study shows that its history cannot be understood without including large population samples for western Europe and Scandinavia.

We confirm the strong differentiation between Europe and Asia within R1a1 [22, 23], and also identify a large group of European chromosomes broadly equivalent to the clade defined by the variant Z282 (Fig. 2) and within this, four sub-haplogroups that encompass almost all central European samples. However, the western-European samples carry not simply the same lineages at lower frequencies, but instead a set of specific sub-haplogroups that are not observed further east (R1a1-GML3*, 2, 8* and 9; Fig. 3b, Table S5). Furthermore, subdividing hg R1a1 has shown that its presence in western Europe is not a simple signature of Viking migration.

Although rare in absolute terms, R1a1-GML3* is the major sub-haplogroup found in Spain, France and Belgium, and also represents 11% of hg R1a1 chromosomes in Great Britain; its distribution seems unrelated to early medieval Viking dispersal, as shown by its complete absence in Scandinavian samples. It is also frequent in Asia (Fig. 3b, Table S5), but because of its paragroup status this does not imply a specific link, and the western-European version seems likely to represent a distinct sub-lineage that could be usefully resolved by additional SNPs.

The previously unrecognised lineage, R1a1-GML2, is deep-rooting (Fig. 2) and western-European specific (Fig. 3b, Table S5), complicating the interpretation of the origins and early spread of hg R1a1. Taken together, its relative frequency in Norway, the British Isles and Iceland, and the structure of the STR network (Fig. 4a), suggest that this lineage is unlikely to owe its distribution to early medieval Viking dispersal, but was spread earlier within Europe’s northwest.

The clade R1a1-GML8 and its sub-haplogroup R1a1-GML9 are absent from our Danish sample, but are the predominant types in Norway, Sweden and Iceland (Fig. 3b; Table S5), and also constitute major sub-haplogroups in Great Britain, marking them as candidate Norse Viking dispersal lineages. R1a-GML9 is seen mostly in peninsular Scandinavia and Great Britain, and its network (Fig. 4c) shows evidence of recent expansion—the greater diversity among Norwegian and Swedish chromosomes is compatible with a migration from Scandinavia to Great Britain, rather than vice versa. The R1a1-GML8* paragroup is also relatively frequent in Norway and the islands of Great Britain, Man and Orkney, and seen in Iceland and in the Inuit. There is a striking predominance of this paragroup over R1a1-GML9 in Orkney and the Isle of Man; this could be influenced by drift, and indeed closely related haplotypes within Orkney support this (Fig. 4b), but it may also suggest a difference in the origin of migrants to Orkney and the Irish Sea (including Isle of Man), compared to the British mainland. Historical sources record close cultural and political relationships between Norway, Orkney and Man in the Viking age [39]. Norwegian political control in Orkney in the form of an earldom began in the ninth century and extended into the fifteenth [40]. The Isle of Man has a more enigmatic and varied history, but like Orkney also faced political influence from Norway, especially after the invasion of Godred Crovan in 1079 CE. Connections between the Isle of Man and Orkney are highly likely owing to their shared Norwegian influence, and may be seen represented in the marriage of Godred Crovan’s son Óláfr Bitlingr with Ingebjorg, daughter of Hákon Paulsson, earl of Orkney [41]. Isotope testing of burials from both Orkney [42] and Man [43] support the notion of migration from Scandinavia, and at least some of it from Norway. In this context, the observations in this study concur with the overall historical picture.

Within the island of Great Britain itself, if hg R1a1 were a reflection of Norse Viking migration, we might expect a higher frequency in regions that show high autosomal contributions [8] from peninsular Scandinavia. However, there is no significant difference between regions with high and low contribution, for either R1a1 as a whole, or for the two candidate Viking sub-lineages identified here. In contrast to this, the proportion of both R1a1 and its two Scandinavian-focused sub-lineages are significantly higher in the region of the Danelaw than outside it, possibly representing a ‘signal’ of this territorial unit that was absent from the autosomal analysis [8]. This might suggest male-specific contribution, or that female mobility has eroded the autosomal signal, for example via patrilocality [44], but work on other MSY lineages within Great Britain is required to address this. Resolving the contribution (or otherwise) of Danes will also require the study of other MSY lineages, since hg R1a1 is present at relatively low frequency in our Danish sample, and the sub-haplogroups found in Denmark are not those that are common in Great Britain. This would face the challenge of distinguishing contributions of Danish Vikings from those of earlier migrants to Britain from the near Continent in the early middle Ages [28]. Additionally, the picture within the Danelaw is complicated by historical factors such as the composition of the Viking Great armies, which were much less homogenous in origin than previously believed [45], as well as by potential large-scale movements, such as the influx of Dublin Vikings in 902 CE. Archaeological investigations have shown diversity of origin within groups buried in the same place [46], which indicates high levels of mobility within the Danelaw.

A better understanding of the histories of Viking migrations and of hg R1a1 sub-haplogroups would be aided by ancient DNA data; this is enabled by the recent publication of whole-genome sequence data on a sample of 442 Viking era genomes (including 297 males) from across the Viking world [47]. International Society of Genetic Genealogy haplogroup names [47] allow the hg R1a1 sub-haplogroups from our data to be identified in the ancient data (Table S6; Fig. S4). As in modern data, proportions of hg R1a1 in Norway (33%) and Sweden (20%) are higher than those in Denmark (8%). Comparisons of the hg R1a1 proportion in modern Norway, Sweden and Denmark with their ancient counterparts shows no significant difference across time for the three populations (*p* > 0.05). Likewise, a population differentiation test of the proportions of the R1a1 sub-lineages in the three populations shows no significant change through time (*p* > 0.05): R1a1-GML8 (including its sub-haplogroup R1a1-GML9) comprised a substantial proportion of hg R1a1 in ancient Norway (71% of R1a1) and Sweden (31% of R1a1). The two sites sampled in Britain lie outside the Danelaw (Dorset and Oxford) and have been interpreted as ‘execution cemeteries’ containing the remains of Viking raiding parties. Haplogroup R1a1 is found in 5/32 males there, and four of these carry R1a1-GML8* or -GML9. Together with the high proportions of these two lineages in ancient Iceland and the Faroe Islands [47], the ancient data support our interpretation of them as Viking expansion lineages originating in peninsular Scandinavia. A broader understanding of the Viking patrilineal contribution to Britain would encompass the full range of MSY haplogroups with more complex histories, and would require the application of a hypothesis-testing approach that considered possible different temporal and geographical origins for lineages [48, 49].

The starting point for this study was an unbiased resource of variants from a set of hg R1a1 Y chromosomes. However, here we have reinstated the bias by cherry-picking SNPs from this set for genotyping. This was a necessary compromise given the expense of large-scale resequencing, but clearly sequencing is preferable. Falling costs in the future should help, but there are also other currently emerging sources of data that could be examined. One is the data due to be produced by the UK’s 100,000 Genomes Project, which will yield a huge number of high-coverage MSY sequences for analysis, but unfortunately without associated fine-scale geographical information. The other is the MSY sequence data generated by commercial providers, either from whole-genome sequences, or from sequence-capture experiments, on behalf of the genetic genealogy community.

## Supplementary information

Supplementary Text

Supplementary Tables

Figures S1-S4
