# Supplementary material for: Subdividing Y-chromosome haplogroup R1a1 reveals Norse Viking dispersal lineages in Britain
Source: Eur J Hum Genet. 2020 Nov 2;29(3):512–23. doi: 10.1038/s41431-020-00747-z (PMC7940619; doi:10.1038/s41431-020-00747-z)
Supplement: Supplementary file 1 — Supplementary Text [file 41431_2020_747_MOESM1_ESM.docx]

**Supplementary Text**

***Ascertainment of hg R1a1 chromosomes for sub-haplogroup analysis***

Six hundred and nineteen DNA samples were subjected to sub-haplogroup analysis (Table S1). These were selected from a number of sample sets, in which one of three different markers had previously been analysed: M17, defining^1^ haplogroup R1a1 (93 samples); SRY_10831.2_, defining hg R1a (345 samples), and M513 (included in the Affymetrix SNP6.0 chip), which is ancestral to SRY_10831.2_ (<http://isogg.org/tree/ISOGG_HapgrpR.html>; 55 samples). In addition, 21 samples were included in which haplogroup status had been ascertained by sequence-capture and next-generation sequencing of 3.7 Mb of MSY^2^. Finally, 105 samples were included after PPY23 Y-STR-based haplogroup prediction using the Haplogroup Predictor tool^3^ available at hprg.com/hapest5/. Of these 105 ‘predicted’ samples, all but eleven proved to be derived for at least one SNP in the sub-haplogroup 10-plex; all of the latter were shown to carry the derived state for the marker M17 using a separate SNaPshot assay.

We note that this strategy ascertains very deep-rooting lineages in the hg R1a1 sub-tree non-uniformly, but given the focus of our study and the apparent great rarity of such lineages (e.g. those derived for SRY_10831.2_ but ancestral for M17), this should not affect our conclusions.

***SNaPshot multiplex for SNP typing***

The 10 chosen SNPs were typed using a multiplex reaction using the Applied Biosystems (AB) SNaPshot kit. Following an initial multiplex PCR to generate amplicons of 245-563 bp encompassing the target SNPs, a second multiplex reaction was performed for single-base extension using fluorescently-labelled terminator nucleotides. The first PCR was done in a volume of 10 µl including 1 µl AB 10x PCR Gold Buffer, AB 25 mM MgCl_2_, 0.16 µl 25 mM dNTPs, and 0.2 µl AB AmpliTaq Gold (5 U/µl). Primers were added as 0.17 µl of 10 µM stock, with the exception of GML5 (20 µM) and M458 (40 µM). 5-50 ng of DNA was added in a volume of 1 µl. PCR was carried out in an MJR Tetrad under the following conditions: 94^°^C for 9 minutes followed by 35 cycles of 30 s at 94^°^C, 30 s at 65^°^C and 1 minute at 72^°^C. Final extension was at 72^°^C for 3 minutes.

PCR products were purified by adding 1.5 µl of 1 U/µl rSAP (Affymetrix), 0.15 µl of 20 U/µl ExoI (Fermentas) and 1.35 µl of 10 x ExoI reaction buffer and incubating for 2 hours at 37^°^C followed by 15 minutes at 80^°^C and 15 minutes at 4^°^C.

Single-base extension (SBE) reactions were carried out in a second reaction, in a 6 µl volume with 1.3 µl AB SNaPshot Multiplex Ready Reaction Mix and 1.3 µl water. SBE primers were added at 10 µM concentration as follows: 0.3 µl of GML1, GML2, GML3, GML4, GML6, GML7 and GML8, 0.6 µl OF GML5, 0.2 µl GML9 and 0.8 µl M458. 1 µl cleaned PCR product was added and the reaction cycled under the following conditions: 35 cycles of 96^°^C for 10 s, 50^°^C for 5 s and 60^°^C for 30 s. SBE products were purified by adding 1 µl of 1 U/µl rSAP (Affymetrix) and the mixture was incubated at 37^°^C for 1 hour, 80^°^C for 15 minutes and 4^°^C for 15 minutes.

PCR products were separated by capillary electrophoresis on an ABI3130xL apparatus (Applied Biosystems) using POP7 polymer and a customised run module and protocol. 1 µl sample DNA was mixed with 9 µl Ultra-pure formamide (AGTC Bioproducts) and 0.2 µl GeneScan LIZ-120 size standard (Applied Biosystems), and samples were denatured for 4 minutes at 96^°^C and placed on ice for 2 minutes before loading. Electropherograms were analysed using GeneMapper software version 4.0 (Applied Biosystems). Custom panel and bin settings used are available on request.

***Additional SNP typing***

In order to place published SNPs^4^ on our phylogenetic tree, we performed PCR-RFLP assays on M558, Z95, Z280, Z284, Z93 in all 27 samples shown in Figure 1, and Sanger-sequenced M417 in four individuals (ork_026m, CEU-NA12155, TSI-NA20796 and ORK-036m). For primer sequences and restriction enzyme information see ref. ^4^.

***Y-STR analysis***

Y-STR haplotype relationships were displayed using median-joining networks^5^ based on 21 STRs (DYS19, DYS389I, DYS389II-I, DYS390, DYS391, DYS392, DYS393, DYS437, DYS438, DYS439, DYS448, DYS456, DYS458, DYS635, YGATAH4, DYS481, DYS533, DYS549, DYS570, DYS576 and DYS643), excluding the bilocal DYS385a,b, within sub-haplogroups R1a1-GML2 and R1a1-GML9, and the paragroup R1a1-GML8*, using Network 5.0 and Network Publisher ([www.fluxus-engineering.com/sharenet.htm](http://www.fluxus-engineering.com/sharenet.htm)). In the case of allele duplications, the smaller allele was considered for network construction, and partial alleles were rounded down to the nearest integral allele. Haplotypes containing null alleles were omitted (two for R1a1-GML2; four for R1a1-GML8*; one for R1a1-GML9). Weighting was done by taking into account the range of mutation rates of the STRs, reflected indirectly by their allele length variances among all chromosomes included in each network^6^.

To estimate the time to the most recent common ancestor (TMRCA) of the European sub-haplogroups R1a1-GML2, R1a1-GML8 (which contains R1a1-GML9) and R1a1-GML9, we used all 21 single-copy Y-STRs and applied the average square distance (ASD) method^7,8^, where the ancestral haplotype in each case was assumed to be the haplotype carrying the most frequent allele at each Y-STR locus. For the calculations, we employed the mean pedigree mutation rate for the 21 Y-STRs based on data available at [www.yhrd.org](http://www.yhrd.org/), namely 3.751 ± 0.694 x 10^-3^ per locus per generation, and a generation time of 30 years^9^. ASD was chosen as a dating method because comparisons of Y-STR and MSY resequencing data^2^ have shown that it outperforms the rho statistic^10^. We chose not to use the ‘evolutionary’ mutation rate^11^ because the same comparisons^2^ have shown that the pedigree mutation rate performs better for young lineages (<10 KYA): our previous resequencing experiments have indicated a SNP-based age for European hg R1a1 Y chromosomes of only 6.0 KYA (95% confidence interval, 4.7–7.3 KYA)^12^. We also tested the effect of using subsets of Y-STRs with slow (DYS438, DYS392, DYS393, DYS437, DYS643, DYS448, DYS390, DYS19, DYS385, DYS385, DYS391) and fast (DYS389I, YGATAH4, DYS533, DYS549, DYS389II, DYS456, DYS635, DYS481, DYS439, DYS458, DYS570, DYS576) mutation rates, using the appropriate mean mutation rates (respectively 1.612 ± 0.234, and 5.919 ± 1.132 x 10^-3^ per locus per generation) from [www.yhrd.org](http://www.yhrd.org/). Population differentiation tests and comparisons based on mean per locus diversity based on STR data were carried out in Arlequin. 3.5^13^.

***Comparing Y-haplogroup distributions with Norwegian+Swedish autosomal contributions***

To compare Y-chromosomal haplogroup distributions with regions in Britain showing high levels of Norwegian plus Swedish autosomal contributions^14^, we made our county-based groupings congruent with the PoBI fineSTRUCTURE-based clusters^14^. This required the combining of some PoBI clusters (e.g. South Pembrokeshire and North Pembrokeshire clusters combined as South Wales), and also the combining of some county-based groupings (e.g. several counties combined into the Central/S.England cluster). These designations are set out in Table S4. To estimate Norwegian plus Swedish autosomal contributions to clusters, we combined the point estimate proportions of the European groups NOR53, NOR61, NOR63, NOR64, NOR71, NOR72, NOR81, NOR85, NOR90, SWE117 and SWE121 as described in the PoBI study^14^. Proportions of hg R1a1 and of R1a1-GML8* + R1a1-GML9 were then compared with high (≥10%) and low (<10%) Norwegian plus Swedish autosomal contribution using a chi square test. The proportions of the same Y-chromosomal lineages were also compared between ‘Danelaw’ and ‘non-Danelaw’ regions, as explained in the main text and set out in Table S4.

**References**

1. Karafet TM, Mendez FL, Meilerman M, Underhill PA, Zegura SL, Hammer MF: New binary polymorphisms reshape and increase resolution of the human Y-chromosomal haplogroup tree. *Genome Res* 2008; **18:** 830-838.

2. Hallast P, Batini C, Zadik D *et al*: The Y-chromosome tree bursts into leaf: 13,000 high-confidence SNPs covering the majority of known clades. *Mol Biol Evol* 2015; **32:** 661-673.

3. Athey TW: Haplogroup prediction from Y-STR values using a Bayesian-allele-frequency approach. *J Genet Geneal* 2006; **2:** 34-39.

4. Underhill PA, Poznik GD, Rootsi S *et al*: The phylogenetic and geographic structure of Y-chromosome haplogroup R1a. *Eur J Hum Genet* 2015; **23:** 124-131.

5. Bandelt H-J, Forster P, Röhl A: Median-joining networks for inferring intraspecific phylogenies. *Mol Biol Evol* 1999; **16:** 37-48.

6. Qamar R, Ayub Q, Mohyuddin A *et al*: Y-chromosomal DNA variation in Pakistan. *Am J Hum Genet* 2002; **70:** 1107-1124.

7. Goldstein DB, Linares AR, Cavalli-Sforza LL, Feldman MW: An evaluation of genetic distances for use with microsatellite loci. *Genetics* 1995; **139:** 463-471.

8. Goldstein DB, Linares AR, Cavalli-Sforza LL, Feldman MW: Genetic absolute dating based on microsatellites and the origin of modern humans. *Proc Natl Acad Sci U S A* 1995; **92:** 6723-6727.

9. Fenner JN: Cross-cultural estimation of the human generation interval for use in genetics-based population divergence studies. *Am J Phys Anthropol* 2005; **128:** 415-423.

10. Saillard J, Forster P, Lynnerup N, Bandelt H-J, Nørby S: mtDNA variation among Greenland Eskimos: the edge of the Beringian expansion. *Am J Hum Genet* 2000; **67:** 718-726.

11. Zhivotovsky LA, Underhill PA, Cinnioglu C *et al*: The effective mutation rate at Y chromosome short tandem repeats, with application to human population-divergence time. *Am J Hum Genet* 2004; **74:** 50-61.

12. Batini C, Hallast P, Zadik D *et al*: Large-scale recent expansion of European patrilineages shown by population resequencing. *Nat Commun* 2015; **6:** 7152.

13. Excoffier L, Lischer HE: Arlequin suite ver 3.5: a new series of programs to perform population genetics analyses under Linux and Windows. *Mol Ecol Resour* 2010; **10:** 564-567.

14. Leslie S, Winney B, Hellenthal G *et al*: The fine-scale genetic structure of the British population. *Nature* 2015; **519:** 309-314.
