# Supplementary material for: Subdividing Y-chromosome haplogroup R1a1 reveals Norse Viking dispersal lineages in Britain
Source: Eur J Hum Genet. 2020 Nov 2;29(3):512–23. doi: 10.1038/s41431-020-00747-z (PMC7940619; doi:10.1038/s41431-020-00747-z)
Supplement: Supplementary file 3 — Figures S1-S4 [file 41431_2020_747_MOESM3_ESM.pdf]

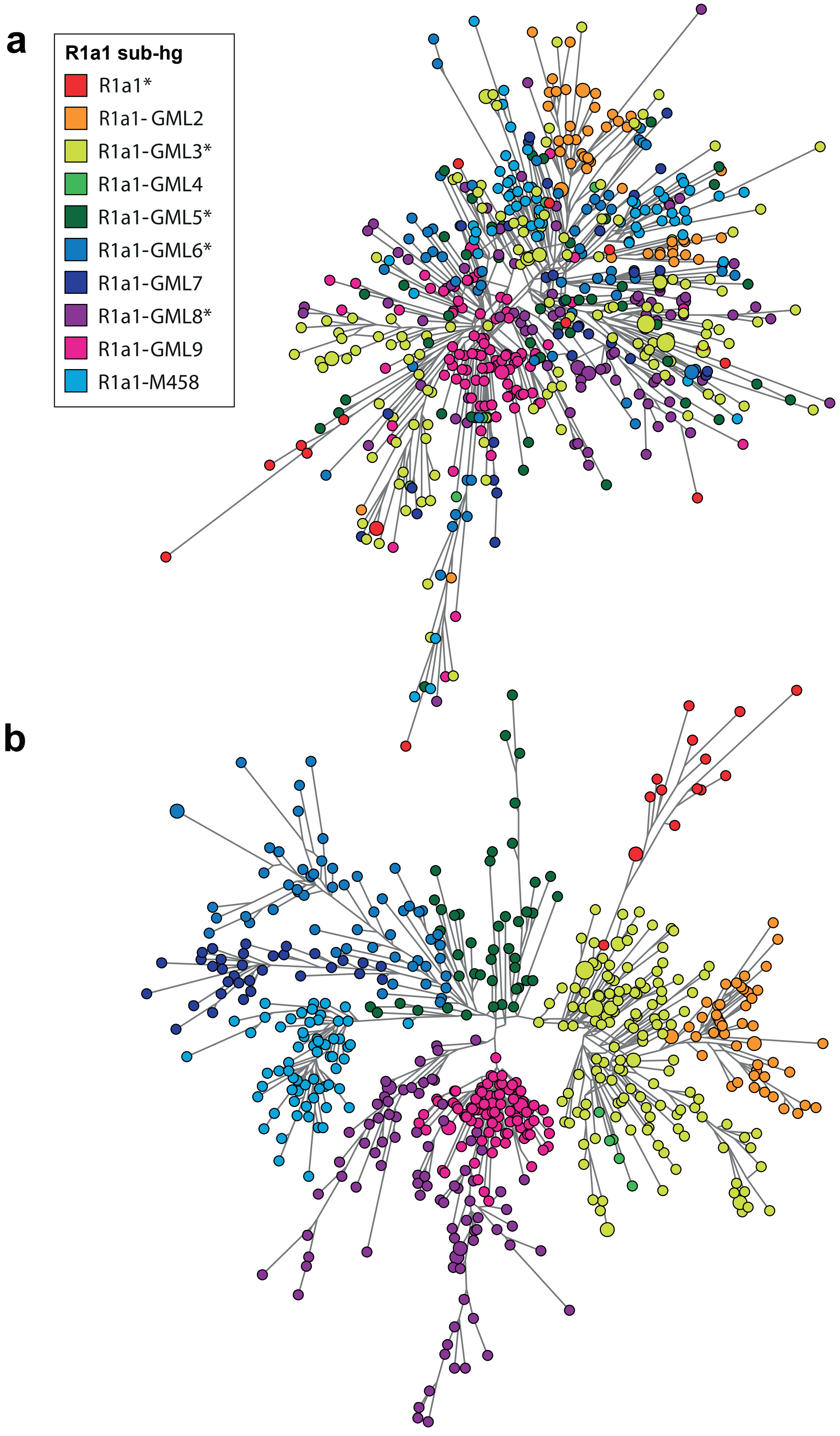

Figure S1: Median-joining networks of hg R1a1 Y-STR haplotypes based on Y-STRs alone, and including SNPs.
Networks based on 21 Y-STRs for hg R1a1 chromosomes based on: a) Y-STRs only, and b) Y-STRs plus the nine SNPs indicated in the key. Circles represent haplotypes, with areas proportional to frequency, and coloured to represent sub-haplogroups as shown in the key. Lines between haplotypes (links) represent STR mutational steps.

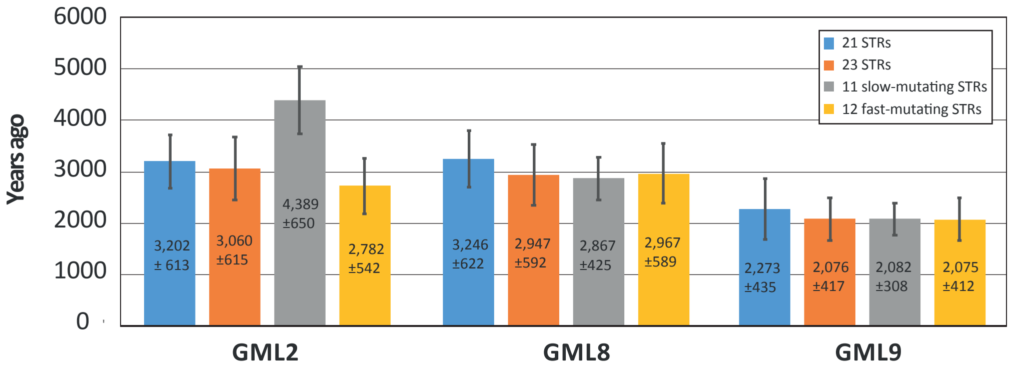

Figure S2: TMRCA estimates of three hg R1a1 sub-lineages based on average square distance (ASD) using different sets of Y-STRs.

Sets of chromosomes being dated here are those represented in Figure 4, except that R1a1-GML8 encompasses both the sub-haplogroup R1a1-GML9 and the paragroup R1a1-GML8*. Sub-sets of Y-STRs are indicated in the key, top right, and described in the Materials & Methods section.

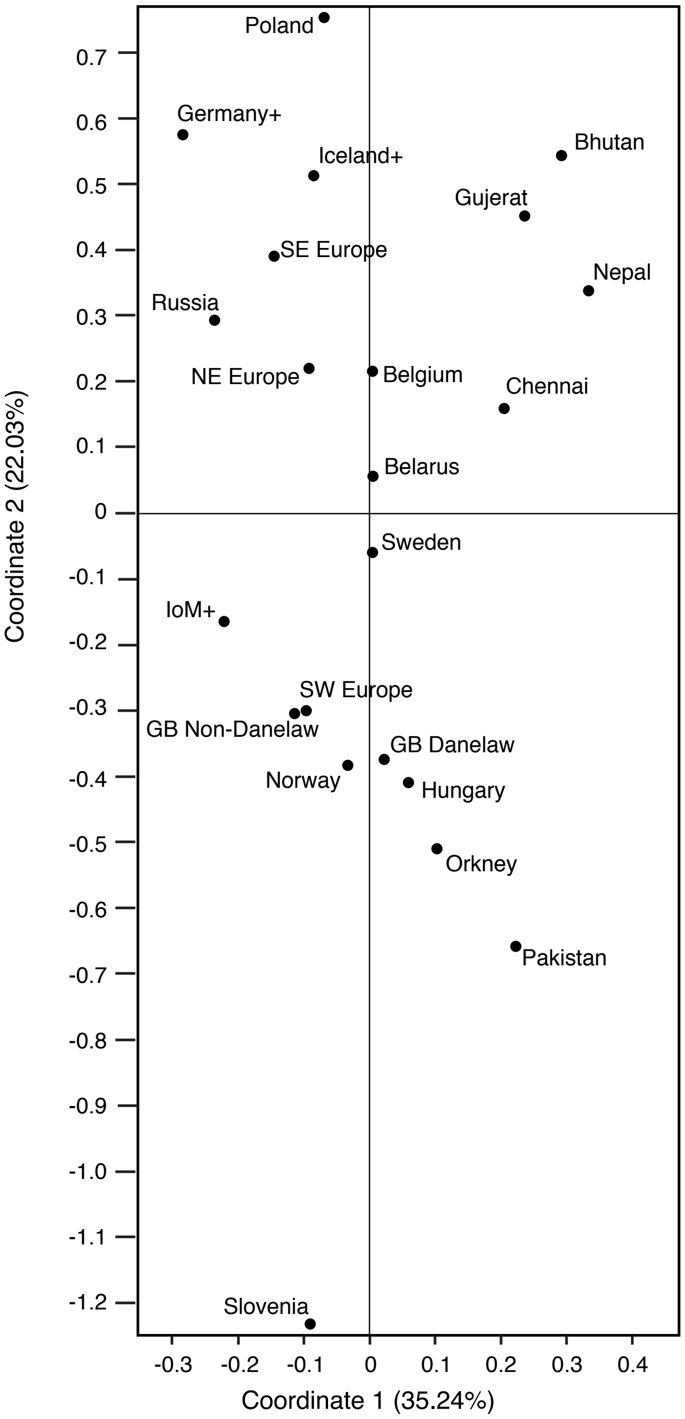

Figure S3: Principal coordinates analysis of populations based on Y-STR haplotypes within hg R1a1.

The minimum population size is 10, and pooling was necessary to achieve this in some cases, as follows: IoM+ = Isle of Man, Scotland and Ireland; Germany+ = Germany and Bavaria; Iceland+ = Iceland and Inuit; SW Europe = France, Spain, Portugal and Italy; SE Europe = Bulgaria, Greece, Turkey, Romania and Serbia; NW Europe = Frisia, Netherlands and Denmark. The two ‘GB’ populations omit the Scottish samples. For some populations, sample size was too small for inclusion and reasonable pooling was not possible.

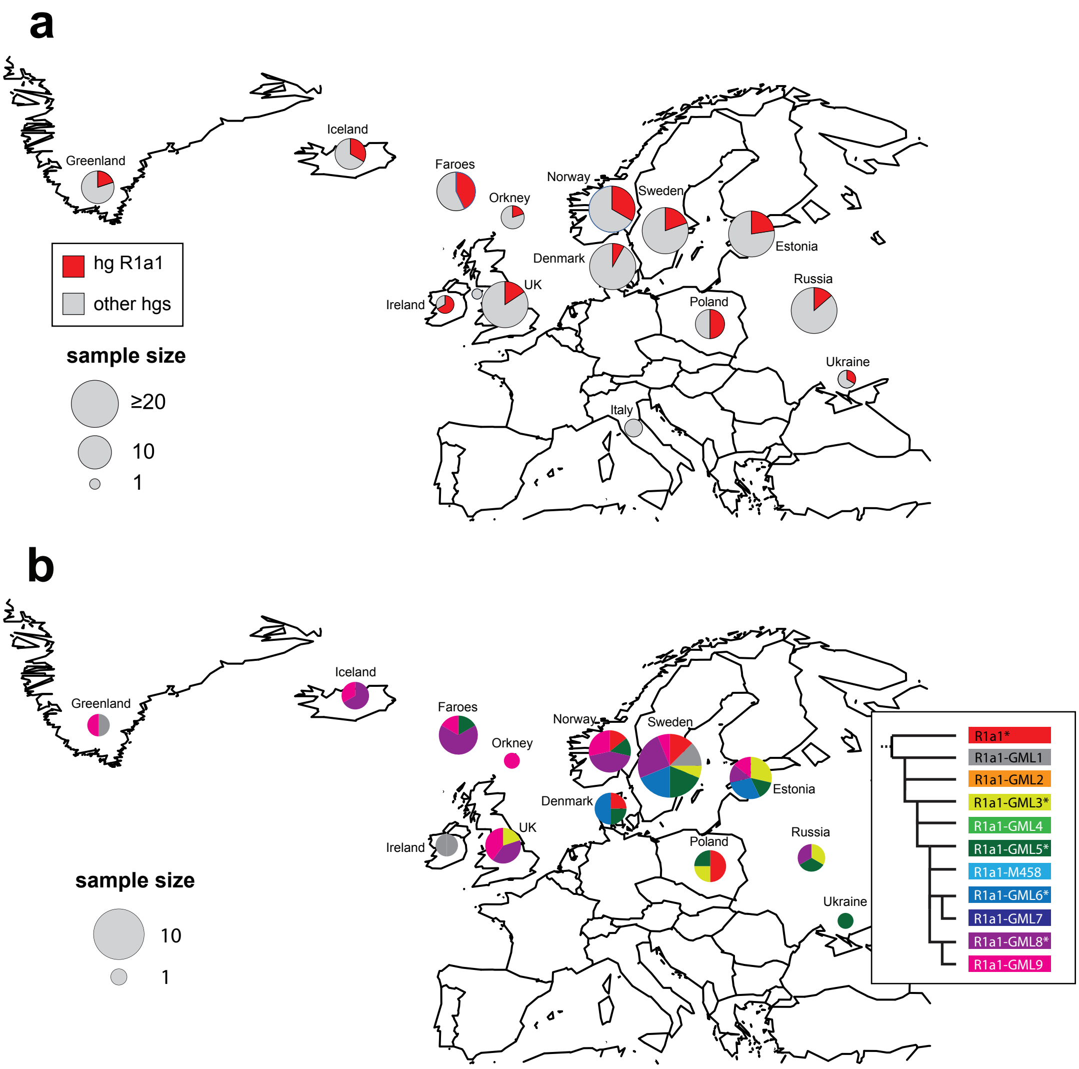

Figure S4: Geographical distributions of haplogroup R1a1 and its sub-haplogroups in ancient DNA.

a) Distribution of haplogroup R1a1 in the analysed samples. Pie-charts indicate populations, with area proportional to sample size up to 20, as indicated in the key, and the red sector showing the proportion of hg R1a1. b) Distribution of sub-haplogroups of R1a1. Populations are represented by pie-charts with area proportional to hg R1a1 sample size up to 20, as indicated in the key, and sectors indicating sub-haplogroup frequencies within R1a1, according to the colour-coded phylogeny top right. Data are from Margaryan et al. (2019).
